# Impact of rapid pathogen detection in ICU patients with suspected pneumonia on antimicrobial therapy: randomized, controlled open-label feasibility trial (IRISPAT-1)

**DOI:** 10.1186/s12890-026-04596-2

**Published:** 2026-08-12

**Authors:** Christopher KC Lai, Alfred Lok Hang Lee, Grace Chung Yan Lui, Jack Zhenhe Zhang, Eddie Leung, Albe Wong, Patricia Leung, Gordon Choi, Eunise Ho, David Ng, Wai Tat Wong, Yu-Yeung Yip, Benjamin Siu, Katy Li, Kwok Ming Ho, Viola CY Chow, Lowell Ling

**Affiliations:** 1 Department of Microbiology, Faculty of Medicine, The Chinese University of Hong Kong, Hong Kong SAR, China; 2 S.H. Ho Research Centre for Infectious Diseases, Faculty of Medicine, The Chinese University of Hong Kong, Hong Kong SAR, China; 3 Department of Microbiology, Prince of Wales Hospital, Hospital Authority, Hong Kong SAR, China; 4 Department of Medicine and Therapeutics, Faculty of Medicine, The Chinese University of Hong Kong, Hong Kong SAR, China; 5 Department of Anaesthesia and Intensive Care, Faculty of Medicine, The Chinese University of Hong Kong, Hong Kong SAR, China; 6 Department of Intensive Care, Prince of Wales Hospital, Hospital Authority, Hong Kong SAR, China

**Keywords:** Community-acquired pneumonia, Hospital-acquired pneumonia, Ventilator-associated pneumonia, Mechanical ventilation, Critically ill

## Abstract

**Background:**

Evidence that multiplex polymerase chain reaction (mPCR) improves antimicrobial therapy and clinical outcomes in the critical care setting is limited. We assessed the impact of rapid mPCR on antibiotic therapy in mechanically ventilated critically ill patients with suspected pneumonia.

**Methods:**

IRISPAT-1 was a single center, randomized, controlled open-label trial designed to determine the feasibility, safety, and potential impact of rapid respiratory pathogen detection on antibiotic therapy in mechanically ventilated critically ill patients with suspected pneumonia. Mechanically ventilated adults with suspected pneumonia and treated with new antibiotics within 24 h were randomized to standard care or mPCR using BioFire^®^ FilmArray^®^ Pneumonia Panel in addition to standard care (FA Pneumonia Panel group). Primary outcome was time interval (hours) to appropriate antimicrobial therapy.

**Results:**

Forty mechanically ventilated patients with suspected pneumonia were randomized (20 = standard care, 20 = FA Pneumonia Panel) between July 7, 2024 and October 4, 2025. Median (IQR) time to appropriate antibiotic therapy was shorter in the FA Pneumonia Panel group compared to the standard care group (7 [5–16] vs. 72 [41–140] hours, HR 3.3; 95%CI [1.6, 6.6], *p* = 0.001). Adherence to treatment algorithm was observed in 90% (18/20) of patients in the FA Pneumonia Panel group. There were no differences in use of broad-spectrum antibiotics, duration of antibiotics, ventilator free days or vasopressor free days and 28-day mortality between treatment groups.

**Conclusion:**

Rapid mPCR with high adherence to antibiotic algorithms shortened time to appropriate antibiotics compared to standard care in mechanically ventilated patients with suspected pneumonia.

**Trial registration:**

Registered at clinicaltrials.gov (NCT06478953) on June 23, 2024.

**Supplementary Information:**

The online version contains supplementary material available at https://doi.org/10.1186/s12890-026-04596-2.

## Introduction

Mechanically ventilated patients with bacterial pneumonia have 20–40% mortality depending on the etiology [1, 2]. However, diagnosing bacterial pneumonia remains challenging since up to 60% of patients yield negative microbiological cultures [3, 4]. Even when causative pathogens are identified, susceptibility testing takes days to complete. Empirical broad-spectrum antimicrobials are frequently administered to critically ill patients with pneumonia to minimize risks of inadequate antimicrobial therapy [5]. But overly-broad antibiotics may be associated with harm and also increase the burden of antimicrobial resistance (AMR) [6–8]. Rapid diagnostic tools are needed to guide selection of appropriate antibiotics which are adequate but as narrowly targeted possible to treat severe bacterial infections.

Observational studies suggest multiplex polymerase chain reaction (mPCR) assays may optimize antibiotic therapy by its high sensitivity and rapid turnover time [9, 10]. However, findings from randomized controlled studies (RCT) have been less promising [11–15]. While some RCTs have shown that mPCR shortens the time to appropriate antibiotics in pneumonia patients, others could not demonstrate any impact of mPCR on antibiotic therapy. However, several methodological limitations in these trials include lack of protocolized antibiotic prescription based on mPCR results, absence of parallel mPCR testing in control groups and the reliance on upper respiratory tract sampling [14, 16, 17]. To date no RCT has specifically evaluated the use of mPCR in critically ill patients on mechanical ventilation, the patient group with the highest mortality risk who most benefits from appropriate antibiotics but also at highest risk of adverse outcomes from inadequate antimicrobial therapy.

To address these gaps, we designed the IRISPAT-1 trial as a feasibility study to determine the impact of rapid respiratory pathogen detection by mPCR on antibiotic therapy in mechanically ventilated critically ill patients with suspected pneumonia. The primary aim was to assess the impact of rapid mPCR with protocolized antibiotic algorithm on time to appropriate antibiotic therapy when compared to standard care. We additionally assessed adherence to protocolized mPCR-guided antibiotic adjustment and its effect on antibiotics usage and patient centered outcomes.

## Methods

### Study design, setting and oversight

IRISPAT-1 was a single center, randomized, controlled open-label trial designed to determine the feasibility, safety, and potential impact of rapid respiratory pathogen detection on antibiotic therapy in mechanically ventilated critically ill patients with suspected pneumonia. The trial was conducted at Prince of Wales Hospital Intensive Care Unit (ICU) in Hong Kong, a 28-bed tertiary ICU that provides regional venous-venous extracorporeal membrane oxygenation (ECMO) service. Our ICU is accredited by the College of Intensive Care Medicine of Australia and New Zealand and achieves an average APACHE-IV standardized mortality ratio of 0.54. Multidisciplinary microbiology rounds are held weekly where microbiologists and intensivists discuss microbiology results and antimicrobial therapy. The trial was overseen by an independent experienced intensivist who reviewed safety data after 20 patients were enrolled. This study was conducted in accordance with the Declaration of Helsinki, approved by the Joint CUHK-NTEC CREC ethics committee (2023.327) and registered on clinicaltrials.gov (NCT06478953) on June 23, 2024. This manuscript followed the CONSORT statement for reporting of RCTs.

### Patient selection and recruitment

Mechanically ventilated adult (aged ≥ 18 years) patients with suspected community acquired, healthcare or ventilator associated pneumonia and treated with new antibiotics within 24 h were eligible for inclusion. Suspected pneumonia was defined as any of purulent sputum, cough, fever, shortness of breath, hypoxia, hypercapnia and abnormal white cell count and chest infiltrates on imaging. Patients were excluded if they were receiving concurrent antibiotic therapy for reasons other than suspected respiratory infection. Other exclusion criteria included aspiration pneumonia, pulmonary tuberculosis, suspicion for *Stenotrophomonas* infection, immunosuppression from long term prednisone of at least 5 mg/day (or equivalent) or chemotherapy or human immunodeficiency viruses or hematological disease since the BioFire^®^ FilmArray^®^ Pneumonia Panel cannot detect anaerobes, opportunistic pathogens such as *Pneumocystis jiroveci* and specific pathogens such as *Stenotrophomonas*. Patients with inadequate high-quality respiratory specimen, antibiotics allergy, pregnancy and limitation of therapy were also excluded. Written consent for study participation was obtained from the patient or their next of kin if they were incapacitated due to critical illness.

### Blinding, randomization & allocation concealment

Patients were randomized with 1:1 ratio to either FilmArray^®^ (FA) Pneumonia Panel or standard care group using a computer-generated sequence. Allocation was concealed in sequentially numbered sealed envelopes, which were opened after recruitment to reveal group assignment. Patients and treating clinical team were unblinded to group assignment because timely adjustment of antimicrobial therapy depended on results of FA Pneumonia Panel. Outcome assessors were blinded to group assignment.

### Respiratory specimen and processing

Immediately after recruitment, tracheal aspirate or bronchoalveolar lavage samples were sent for both conventional microbiological cultures and mPCR. All microbiological samples (including mPCR) were processed in the local microbiology laboratory to comply with local infection control policy.

### BioFire^®^ FilmArray^®^ Pneumonia Panel

BioFire^®^ FilmArray^®^ Pneumonia Panel is a mPCR based in vitro assay which identifies 8 viral and 18 bacterial common pathogens in tracheal aspirate and BAL samples (Supplementary Methods). It provides semi-quantitative counts for 15 bacterial species to help differentiate pathogen from colonizer. In addition, 7 common antimicrobial resistance genes: carbapenamases (IMP, KPC, NDM, OXA-48-like), ESBL (CTX-M) and MRSA (mecA/C and MREJ) are tested.

### Standard care group

Patients in the standard care group received empirical antibiotic therapy based on clinical judgment and subsequent targeted antibiotic therapy guided by conventional microbiological gram stain and cultures and procalcitonin protocol (Supplementary Methods). Use of the narrowest-spectrum antibiotics was based on culture sensitivity. Standard investigations for mechanically ventilated patient with suspected pneumonia included two sets of blood cultures, alternate day procalcitonin, respiratory specimen of tracheal aspirate or bronchoalveolar lavage for bacterial gram stain and cultures. Respiratory viral PCR and urine streptococcus and legionella antigen tests were performed in suspected CAP. BioFire^®^ FilmArray^®^ Pneumonia Panel assays were performed on concurrent respiratory samples in the standard care group and used by the Antimicrobial Stewardship Review Panel to assess study outcomes at the conclusion of the study. mPCR results were blinded to the clinical team unless a statutory notifiable pathogen such as *Legionella pneumophila* and Middle East Respiratory Syndrome coronavirus was detected.

### FA Pneumonia Panel group

Patients in the FA Pneumonia Panel group had BioFire^®^ FilmArray^®^ Pneumonia Panel assays performed within 24 h of randomization in addition to standard care. Results were sent to the clinical team within 6 h of sample processing, and antimicrobial therapy were adjusted according to a pre-determined treatment algorithm and antimicrobial guidelines (Supplementary Methods). The treatment algorithm and antimicrobial guidelines were based on the latest hospital antibiogram, procalcitonin protocol, and consensus recommendations from microbiologists, infectious disease experts and intensivists. Clinical rationales were recorded if the clinical team did not follow the pre-determined antimicrobial algorithm after mPCR results were reported.

### Antimicrobial stewardship review panel

The Antimicrobial Stewardship Review Panel consisted of a microbiologist, an infectious diseases specialist and an intensivist that were blinded to study group allocation. At the study conclusion, the panel reviewed the clinical history, imaging, laboratory data, conventional microbiological gram stain and culture, BioFire^®^ FilmArray^®^ Pneumonia Panel results and clinical response to determine diagnosis, causative respiratory pathogen and appropriateness of antimicrobial therapy for each patient. Confirmed pneumonia was established when patient was diagnosed with pneumonia and at least one causative pathogen was identified. A clinical diagnosis of bacterial pneumonia could be established even if a causative respiratory pathogen was not identified. Alternatively, non-infective diagnoses such as pulmonary oedema or secondary acute respiratory distress syndrome were attributed as the cause of respiratory failure if pneumonia was not present. The combination of pneumonia with other diagnoses was permitted.

Appropriate antimicrobial therapy in patients with bacterial pneumonia was defined as use of the narrowest spectrum antimicrobial agent relative to the pathogen’s sensitivity pattern or clinical context if pathogens were not isolated. For patients who were considered not to have respiratory infection after retrospective review, discontinuation of all antimicrobials was required to be deemed as appropriate antimicrobial therapy.

### Outcomes measures

The primary outcome was time interval (hours) to appropriate antimicrobial therapy, defined as the difference in time from randomization to earliest time that appropriate antimicrobial therapy is achieved. Time interval of zero was assigned for patients who were already on appropriate antimicrobial therapy at recruitment. This outcome was censored at 7 days after randomization [15].

Secondary outcomes included proportion of appropriate antimicrobial therapy, proportion of broad-spectrum antibiotics usage, proportion of patients requiring reinitiation or escalation of antibiotics after discontinuation or de-escalation at 48 h after randomization, time to causative pathogen detection, duration of antibiotic therapy, ventilator free days, vasopressor free days and all-cause 28-day mortality. Broad-spectrum antibiotics included carbapenem, tigecycline, ceftolozane-tazobactam, ceftaroline, ceftazidime/avibactam, linezolid, vancomycin, daptomycin, aztreonam or cefiderocol. Since only 25% of patients were treated with broad-spectrum antibiotics at recruitment, a post-hoc analysis was performed to assess the difference in use of more general broad-spectrum antibiotics with activity against *Pseudomonas spp.* or methicillin-resistant *Staphylococcus aureus* in patients initially treated with these antibiotics [8]. Time to causative pathogen detection was measured as the time interval (hours) from randomization to the earliest time that all causative pathogens were confirmed by microbiological tests made available to the clinical team (censored at 7 days). This included pathogens from non-pulmonary sources of infection. If only non-infective respiratory diagnoses were identified, time to detection of causative pathogen was defined as the time interval between randomization and reporting time of negative respiratory tract cultures. Respiratory pathogens assessed as colonizers were not considered diagnostic. The duration of antibiotic therapy was calculated as the number of antibiotic days within 28 days of randomization. Ventilator and vasopressor free days were determined by counting the number of days that the patient was alive and free from mechanical ventilatory support or vasopressor therapy, respectively.

Diagnostic performance of rapid mPCR in addition to standard care was assessed as an “add on test” and compared to standard care alone [18]. The causative respiratory pathogen as determined by the Antimicrobial Stewardship Review Panel was used as the gold standard. The Antimicrobial Stewardship Review Panel classified non-causative pathogen as colonizers (false positive detection) by considering the clinical context. Diagnostic performance was assessed by sensitivity, specificity, positive predictive value, negative predictive value and area under the receiver operating characteristic (AUC) curve. We additionally evaluated the concordance between mPCR and respiratory tract microbiological culture and viral PCR using Cohen’s 𝜅. Concordance was achieved when pathogens detected by mPCR and respiratory tract cultures and viral PCR were the same.

### Statistical analysis

Intention to treat analysis was used to assess differences in outcomes between treatment groups. Differences in proportions between groups were tested with Chi-Square test, and Mann-Whitney U test was used to assess differences in continuous outcomes. Prespecified subgroup analysis was performed on patients initially treated with at least one broad-spectrum antimicrobial drug at time of recruitment. Differences in time to appropriate antibiotics and pathogen detection were assessed by Cox-proportional hazards model with hazard ratios (HR). Variance was reported with 95% confidence intervals and significance level was set at α = 0.05 without adjustment for multiplicity. All statistical analyses were performed in R (version 2024.04.2).

### Sample size

The FLAGSHIP II trial found that use of mPCR in patients with suspected pneumonia reduced the time of inappropriate antibiotics from 85.7 to 47.1 h when compared to standard care [15]. Based on this observed effect size (Cohen’s d = 0.7), a pilot study sample size of at least 10 patients per treatment arm was needed to inform the design of a larger confirmatory trial with 90% power and α of 0.05 [19]. We estimated that a total of 40 patients was sufficient for this pilot study.

## Results

### Study population

**Fig. 1:**
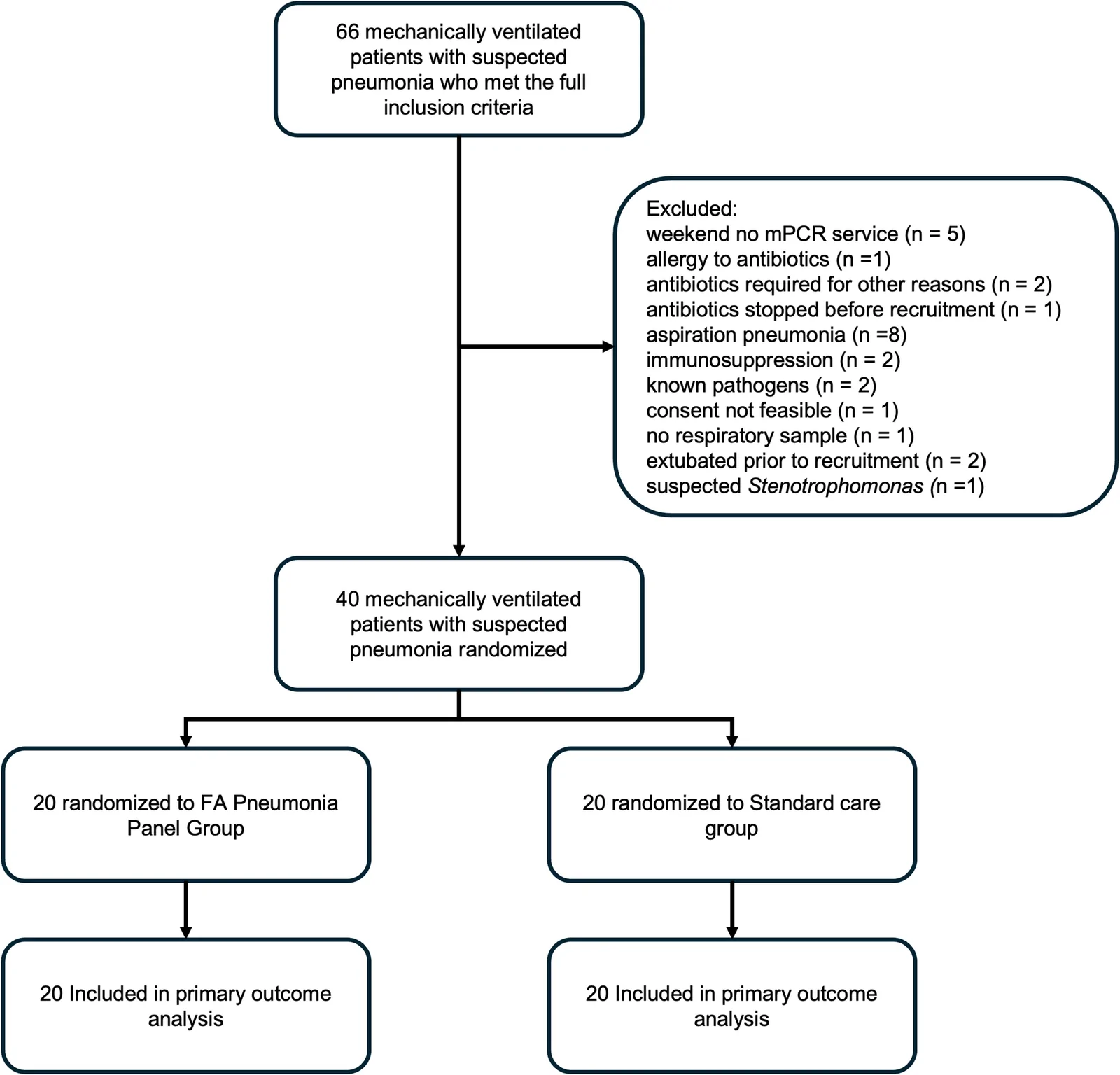
Study flow. mPCR, multiplex polymerase chain reaction

A total of 40 mechanically ventilated patients with suspected pneumonia were randomized between July 7, 2024 and October 4, 2025 (Fig. 1). Follow-up was complete and concluded on November 1, 2025. Most baseline demographics and characteristics were similar between standard care and FA Pneumonia Panel groups (Table 1). The only differences were higher median (IQR) APACHE IV score (94 [85–114] vs. 68 [54–91], *p* = 0.034) and slightly shorter median days on mechanical ventilation prior to recruitment (0 [0–0] vs. 1 [0–4], *p* = 0.019) in standard care group compared to FA Pneumonia Panel group, respectively. 

**Table 1:** Demographics and characteristics

	Standard Care *n* = 20	FA Pneumonia Panel *n* = 20	*p*
Age, years	62 (51–68)	64 (54–71)	0.675
Male Sex	14 (70)	13 (65)	1
Charlson Comorbidity Index	3 (2–4)	4 (2–5)	0.603
Admission Source			
Emergency Department	8 (40)	6 (30)	0.075
Hospital Ward	11 (55)	7 (30)	
Operation Theatre	1 (5)	7 (30)	
P_a_O_2_/F_i_O_2_, mmHg	115 (92–180)	204 (124–263)	0.133
APACHE IV	94 (85–114)	68 (54–91)	0.034
SOFA	8 (7–10)	7 (5–8)	0.539
Procalcitonin, ng/mL	0.59 (0.27–16.28)	0.16 (0.05–1.13)	0.069
Type of Suspected Pneumonia (%)			
CAP	14 (70)	10 (50)	0.208
HAP	3 (15)	2 (10)	
VAP	3 (15)	8 (40)	
Broad-spectrum Antibiotics (%)	5 (25)	5 (25)	1
Anti-pseudomonal or Anti-MRSA Antibiotics (%)	14 (70)	13 (65)	1
Days on Mechanical Ventilation	0 (0–0)	1 (0–4)	0.019
Dialysis (%)	5 (25)	2 (10)	0.408
Vasopressor (%)	16 (80)	11 (55)	0.177

All values are expressed in median (interquartile range) unless otherwise specified

*APACHE IV* Acute Physiology And Chronic Health Evaluation IV, *CAP* community acquired pneumonia, *HAP* healthcare-associated pneumonia, *MRSA* methicillin-resistant *Staphylococcus aureus,* *P*_a_*O*_2_/*F*_i_*O*_2_ Partial pressure of arterial oxygen/Fraction of Inspired Oxygen, *SOFA* sequential organ failure assessment score, *VAP* ventilator-associated pneumonia

### Interventions and study procedure adherence

Patient specimen sent for mPCR test and conventional microbiological culture included tracheal aspirates (95%) and bronchial aspirate/alveolar lavage (5%) samples. Respiratory tract cultures and mPCR test were obtained prior to antibiotic administration in 62.5% and 17.5% of patients, respectively.

All patients in the FA Pneumonia Panel group had mPCR tests reported within 24 h of recruitment. One patient in the standard care group did not have mPCR test due to missing sample. Positive mPCR for *Legionella pneumophila* from one patient in the standard care group was unblinded to comply with public health policy. Adherence to treatment algorithm was observed in 90.0% (18/20) of patients in the FA Pneumonia Panel group. The two reasons for non-adherence were due to identification of immunosuppression in one patient after randomization and concern with potential AMR not covered by gene testing in a patient receiving ECMO.

### Diagnosis, causative pathogen and appropriate antibiotics

The Antimicrobial Stewardship Review Panel reached consensus on the diagnoses, causative pathogens and appropriate antibiotics for all patients. Overall, 14 (70%) patients in each group were classified as pneumonia. Additionally, 20% (4/20) of patients in the standard care group had other diagnoses concurrent with pneumonia (Table 2 and Supplementary Table 1). Amongst patients diagnosed with pneumonia, causative pathogens were identified in 72.2% (13/18) of the standard care group and 85.7% (12/14) of the FA Pneumonia Panel group. The panel determined that antibiotics were required in 85.0% (17/20) of standard care group and 70% (14/20) of FA Pneumonia Panel groups. Two patients in the standard care group (2/20) compared to none in the FA Pneumonia Panel group (0/20) were on appropriate antibiotics at recruitment.

**Table 2:** Diagnosis and cause of respiratory failure

Diagnosis	Standard Care *n* = 20	FA Pneumonia Panel *n* = 20
Pneumonia only (CAP, HAP or VAP)	14	14
Pneumonia and Acute Pulmonary Edema	3	0
Pneumonia and *Clostridium difficile* Colitis	1	0
Acute Pulmonary Edema and *Salmonella enterica* Gastroenteritis	0	1
Acute Pulmonary Edema	2	0
Asthma and Upper Respiratory Tract Infection	0	1
Massive Hemoptysis	0	2
Sputum Retention	0	2

Diagnosis and causes of respiratory failure as determined by Antibiotic Stewardship Review Panel

*CAP* community-acquired pneumonia, *HAP* healthcare-associated pneumonia, *VAP* ventilator-associated pneumonia

### Primary outcome

**Fig. 2:**
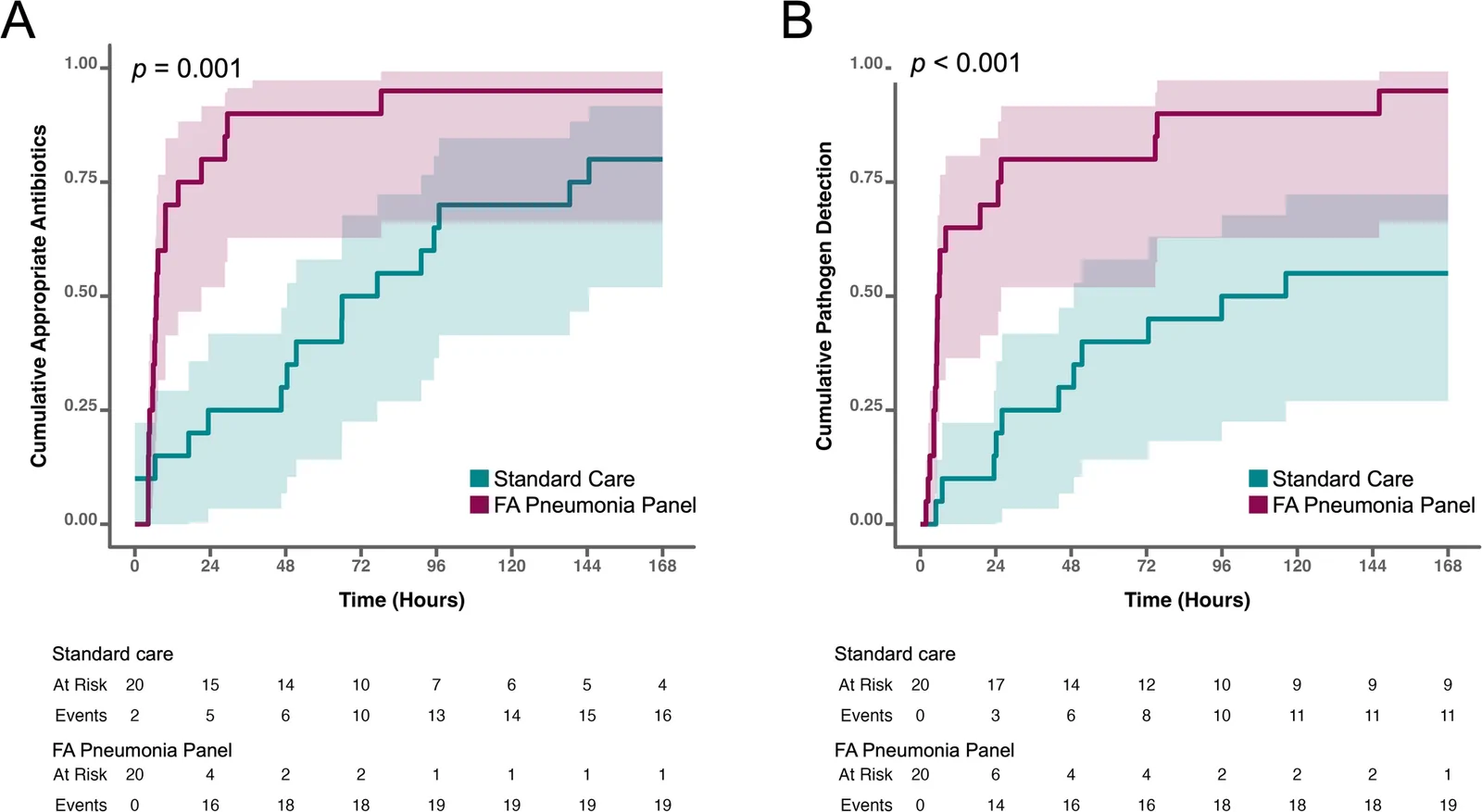
Time to appropriate antibiotics and pathogen detection. Figure shows the time to (**A**) appropriate antibiotics and (**B**) pathogen detection from randomization in FA Pneumonia Panel and standard care group. Appropriate antibiotics and pathogen was determined by the Antimicrobial Stewardship Review Panel at the conclusion of the study. Both outcomes were censored at 7 days after randomization

Appropriate antibiotic therapy was achieved within 7 days in 80% (16/20) of patients in the standard care group and 95% (19/20) of patients in the FA Pneumonia Panel group. The proportionality of the hazards assumption was met (*p* = 0.26). Median (IQR) time to appropriate antibiotic therapy was shorter in the FA Pneumonia Panel group compared to the standard care group (7 [5–16] vs. 72 [41–140] hours, HR 3.3 [95%CI: 1.6, 6.6], *p* = 0.001) (Table 3; Fig. 2).

**Table 3:** Primary and secondary outcomes

	Standard Care *n* = 20	FA Pneumonia Panel *n* = 20	Hazard Ratio or Absolute Risk Difference or Mean Difference (95%CI)	*p*
Primary Outcome				
Time to Appropriate Antibiotic Therapy (Hours)	72 (41–140)	7 (5–16)	3.3 (1.6, 6.6)^#^	0.001
Secondary Outcomes				
Time to Pathogen Detection (Hours)	106 (40–168)	6 (5–25)	4.0 (1.9–8.7) ^#^	< 0.001
Duration of Antibiotics (Days)	9 (4–12)	7 (5–10)	0 (-5, 4)	0.705
Broad-spectrum antibiotics at 48 h* (%)	5 (25)	4 (20)	-5 (-36, 26)*	1
Reinitiation or Escalation of Antibiotics after 48 h (%)	1 (5)	1 (5)	0 (-0.1, 0.1)*	1
ICU Mortality (%)	4 (20)	0 (0)	-20 (-43, 2)	0.106
ICU LOS (Days)	11 (8–16)	12 (8–16)	-4 (-15, 7)	0.765
28-Day Mortality (%)	6 (30)	1 (5)	-25 (-52, 2)*	0.091
Ventilator Free Days	22 (19–24)	23 (20–24)	2 (-1, 5)	0.333
Vasopressor Free Days	25 (23–26)	27 (25–28)	1 (0, 3)	0.066

All values are expressed in median (interquartile range) unless otherwise specified

^#^Hazard ratio

*Absolute Risk Difference

### Secondary outcomes

Median time to pathogen detection was shorter in the FA Pneumonia Panel group compared to the standard care group (6 [5–25] vs. 106 [40–168] hours, HR 4.0 [95%CI: 1.9–8.7], *p* < 0.001). There was no difference in duration of antibiotics between FA Pneumonia Panel group and standard care group (7 [5–10] vs. 9 [4–12] days, *p* = 0.705). Similarly, there was no difference in proportion of broad-spectrum antibiotic usage between FA Pneumonia Panel group compared to standard care group (20% vs. 25%, absolute risk difference − 5% [95%CI: -36, 26], *p* = 1) at 48 h after randomization. However, among the subgroup of patients receiving activity against *Pseudomonas* spp. or methicillin-resistant *Staphylococcus aureus* at time of recruitment, the FA Pneumonia Panel group demonstrated lower usage of these antibiotics at 48 h compared to the standard care group (39% vs. 86%; absolute risk difference − 50% [95%CI: -87, -8], *p* = 0.032). There were no differences in reinitiation or escalation of antibiotics at 48 h after recruitment, ICU mortality, ICU LOS, ventilator free days or vasopressor free days and 28-day mortality (Table 3).

### Diagnostic performance

Addition of mPCR to standard care improved diagnostic sensitivity for respiratory pathogens from 0.45 (95%CI: 0.27, 0.65) to 0.85 (95%CI: 0.75, 1.00) compared to standard care alone (Supplementary Table 2). Specificity was similar between mPCR with standard care (0.91 [95%CI: 0.71, 096]) and standard care alone (0.92 [95%CI: 0.75, 1.00]). AUC of mPCR with standard care (AUC 0.88 [95%CI: 0.78, 0.99]) was higher than standard care alone (AUC 0.69 [95%CI: 0.56, 0.82]) (*p* = 0.028). Agreement between mPCR and respiratory tract cultures and viral PCR was 59.0% (Cohen’s 𝜅 = 0.52, *p* < 0.001).

## Discussion

In this feasibility study on mechanically ventilated critically ill patients with suspected pneumonia, use of rapid mPCR with a protocolized antibiotic algorithm compared to standard care resulted in shorter time to appropriate antibiotics and pathogen detection. Adherence to protocolized mPCR guided antibiotic adjustment was high. However, there was no difference in use of broad-spectrum antibiotics, duration of antibiotic therapy, days free from mechanical ventilation or vasopressors and 28-day mortality.

Our study demonstrated that mPCR significantly reduced time to appropriate antibiotics from 72 h to 7 h over standard care in mechanically ventilated patients with suspected pneumonia. This finding contrasts to recent trials that have failed to demonstrate similar impact of mPCR on antibiotic therapy in patients with pneumonia [14, 20]. A key methodological difference is our approach to antibiotic adjustments. Unlike other studies where recommendations were provided but adjustments themselves were neither protocolized nor enforced, we achieved 90% adherence to a pre-determined, protocolized antibiotic algorithm. Indeed, even with conventional bacterial cultures, clinicians commonly do not de-escalate antibiotics despite knowing bacterial susceptibilities [21]. These findings highlight that overcoming behavioral barriers to optimize antibiotic therapy remains a persistent challenge in appropriate use of microbiological tests, whether established or new [22].

Rapid and accurate mPCR results are essential for clinical translation. Our results show that time to pathogen detection was shortened from 106 h to 6 h when mPCR was used in addition to standard care. Several factors contributed to this finding. First, the BioFire^®^ FilmArray^®^ Pneumonia Panel has very high sensitivity for the pathogens tested in the assay [9]. Second, only high quality lower respiratory tract samples were used for mPCR testing [14]. Third, the trial was designed to exclude patients at high risk of opportunistic infections and pathogens such as *Stenotrophomonas* to minimize the risk of false negative mPCR results.

No differences were observed in overall broad-spectrum antibiotic use or total antibiotic duration when comparing use of mPCR in addition to standard care. These findings align with recent studies that also demonstrated high adherence to a treatment algorithm utilizing rapid mPCR and procalcitonin [12, 13, 17]. Our findings can be attributed to several key factors. Firstly, both study groups had high proportions of confirmed bacterial pneumonia. The greatest reduction in antibiotic duration derived from mPCR may be in patients not requiring antibiotics such as pulmonary oedema that were initially treated for suspected pneumonia. Future trials may consider studying the utility of mPCR in this specific group of patients with high pre-test probability of non-infectious causes of respiratory failure. Secondly, some critically ill patients initially treated for suspected pneumonia may subsequently have extra-pulmonary bacterial infections (such as salmonella gastroenteritis) necessitating antibiotic therapy. Nevertheless, unlike most trials on respiratory mPCR, we stringently excluded patients that had other reasons for antibiotic therapy at recruitment. Thirdly, only 25% of patients received broad-spectrum antibiotics at the time of recruitment, which may have limited the potential for antibiotic de-escalation guided by mPCR.

Currently no trial has shown that mPCR improves survival from pneumonia, but a recent meta-analysis on bloodstream infections concluded that antimicrobial stewardship is essential to maximize the efficacy of mPCR in improving patient-centered outcomes [13, 23, 24]. Our results show that strict adherence to antibiotic protocol guided by mPCR is feasible and improves time to appropriate antibiotics in mechanically ventilated with suspected pneumonia. Larger confirmatory trials are needed to see if this approach improves patient outcomes.

This study had several limitations. First, this was a single center feasibility study. Second, our cohort’s low prevalence of multidrug-resistant pathogens and predominance of CAP limits the generalizability of findings to settings with higher AMR or nosocomial pneumonia [25]. Third, patients with immunosuppression were excluded in this study. A recent retrospective study found that mPCR may still be helpful in immunocompromised patients with suspected pneumonia, because a significant portion of these patients are caused by the same common bacterial pathogens in immunocompetent hosts [26, 27]. Fourth, respiratory tract microbiological culture was sent after antibiotic administration in 37% of patients, which may have reduced its diagnostic yield.

## Conclusion

In mechanically ventilated patients with suspected pneumonia, use of rapid mPCR with high adherence to an antibiotic algorithm shortened time to appropriate antibiotics and pathogen detection compared with standard care. Larger confirmatory trials are needed to determine if this approach improves patient-centered outcomes.

## Supplementary Information

Supplementary Material 1.

Supplementary Material 2.

## Data Availability

The datasets used and/or analysed during the current study are available from the corresponding author on reasonable request and approval from ethics committees.

## Abbreviations

APACHE: Acute Physiology and Chronic Health Evaluation
AMR: Antimicrobial resistance
CAP: Community-acquired pneumonia
ECMO: Extra-corporeal membrane oxygenation
FA: FilmArray^®^
HAP: Healthcare-associated pneumonia
ICU: Intensive care unit
IQR: Interquartile range
LOS: Length of stay
mPCR: Multiplex polymerase chain reaction
RCT: Randomized controlled trial
VAP: Ventilator-associated pneumonia

## References

[1] Hraiech S, Alingrin J, Dizier S, Brunet J, Forel JM, La Scola B, et al. Time to intubation is associated with outcome in patients with community-acquired pneumonia. PLoS ONE. 2013;8(9):e74937. 10.1371/journal.pone.0074937 PMC3777932 24069367

[2] Talbot GH, Das A, Cush S, Dane A, Wible M, Echols R, et al. Evidence-Based Study Design for Hospital-Acquired Bacterial Pneumonia and Ventilator-Associated Bacterial Pneumonia. J Infect Dis. 2019;219(10):1536–44. 10.1093/infdis/jiy578 PMC6562159 30649434

[3] Kharel S, Bist A, Mishra SK. Ventilator-associated pneumonia among ICU patients in WHO Southeast Asian region: A systematic review. PLoS ONE. 2021;16(3):e0247832. 10.1371/journal.pone.0247832 PMC7942996 33690663

[4] Skrupky LP, McConnell K, Dallas J, Kollef MH. A comparison of ventilator-associated pneumonia rates as identified according to the National Healthcare Safety Network and American College of Chest Physicians criteria. Crit Care Med. 2012;40(1):281–4. 10.1097/CCM.0b013e31822d7913 21926609

[5] Ling L, Wong WT, Lipman J, Joynt GM. A Narrative Review on the Approach to Antimicrobial Use in Ventilated Patients with Multidrug Resistant Organisms in Respiratory Samples-To Treat or Not to Treat? That Is the Question. Antibiot (Basel). 2022;11(4):452. 10.3390/antibiotics11040452 PMC9031060 35453203

[6] Michael CA, Dominey-Howes D, Labbate M. The antimicrobial resistance crisis: causes, consequences, and management. Front Public Health. 2014;2:145. 10.3389/fpubh.2014.00145 PMC4165128 25279369

[7] Armand-Lefevre L, Angebault C, Barbier F, Hamelet E, Defrance G, Ruppe E, et al. Emergence of imipenem-resistant gram-negative bacilli in intestinal flora of intensive care patients. Antimicrob Agents Chemother. 2013;57(3):1488–95. 10.1128/AAC.01823-12 PMC3591916 23318796

[8] Zhang JZ, Chan CH, Chang LC, Chiu LCS, Ng PY, Dharmangadan M, et al. Regional Adherence to Early Sepsis Management Bundle and Associated Mortality in Hong Kong Between 2009–2018. Open Forum Infect Dis. 2026;13(1):ofaf752. 10.1093/ofid/ofaf752 PMC12759782 41488700

[9] Buchan BW, Windham S, Balada-Llasat JM, Leber A, Harrington A, Relich R, et al. Practical Comparison of the BioFire FilmArray Pneumonia Panel to Routine Diagnostic Methods and Potential Impact on Antimicrobial Stewardship in Adult Hospitalized Patients with Lower Respiratory Tract Infections. J Clin Microbiol. 2020;58(7):e00135-20. 10.1128/JCM.00135-20 PMC7315039 32350045

[10] Peiffer-Smadja N, Bouadma L, Mathy V, Allouche K, Patrier J, Reboul M, et al. Performance and impact of a multiplex PCR in ICU patients with ventilator-associated pneumonia or ventilated hospital-acquired pneumonia. Crit Care. 2020;24(1):366. 10.1186/s13054-020-03067-2 PMC7303941 32560662

[11] Poole S, Tanner AR, Naidu VV, Borca F, Phan H, Saeed K, et al. Molecular point-of-care testing for lower respiratory tract pathogens improves safe antibiotic de-escalation in patients with pneumonia in the ICU: Results of a randomised controlled trial. J Infect. 2022;85(6):625–33. 10.1016/j.jinf.2022.09.003 36096312

[12] Fartoukh M, Nseir S, Megarbane B, Cohen Y, Lafarge A, Contou D, et al. Respiratory multiplex PCR and procalcitonin to reduce antibiotic exposure in severe SARS-CoV-2 pneumonia: a multicentre randomized controlled trial. Clin Microbiol Infect. 2023;29(6):734–43. 10.1016/j.cmi.2023.01.009 PMC9847221 36681325

[13] Voiriot G, Argaud L, Cohen Y, Tuffet S, Chauvelot L, Souweine B, et al. Combined use of a multiplex PCR and serum procalcitonin to reduce antibiotic exposure in critically ill patients with community-acquired pneumonia: the MULTI-CAP randomized controlled trial. Intensive Care Med. 2025;51(8):1417–30. 10.1007/s00134-025-08014-9 40663137

[14] Abelenda-Alonso G, Calatayud L, Rombauts A, Meije Y, Oriol I, Sopena N, et al. Multiplex real-time PCR in non-invasive respiratory samples to reduce antibiotic use in community-acquired pneumonia: a randomised trial. Nat Commun. 2024;15(1):7098. 10.1038/s41467-024-51547-8 PMC11330507 39154071

[15] Darie AM, Khanna N, Jahn K, Osthoff M, Bassetti S, Osthoff M, et al. Fast multiplex bacterial PCR of bronchoalveolar lavage for antibiotic stewardship in hospitalised patients with pneumonia at risk of Gram-negative bacterial infection (Flagship II): a multicentre, randomised controlled trial. Lancet Respir Med. 2022;10(9):877–87. 10.1016/S2213-2600(22)00086-8 35617987

[16] Markussen DL, Serigstad S, Ritz C, Knoop ST, Ebbesen MH, Faurholt-Jepsen D, et al. Diagnostic Stewardship in Community-Acquired Pneumonia With Syndromic Molecular Testing: A Randomized Clinical Trial. JAMA Netw Open. 2024;7(3):e240830. 10.1001/jamanetworkopen.2024.0830 PMC10918504 38446481

[17] Sabate-Elabbadi A, Mekontso-Dessap A, Lionnet F, Santin A, Verdet C, Woerther PL, et al. Combined use of respiratory multiplex PCR and procalcitonin to reduce antibiotic exposure in sickle-cell adult patients with acute chest syndrome (The ANTIBIO-STA study): a randomised, controlled, open-label trial. Lancet Reg Health Eur. 2025;51:101234. 10.1016/j.lanepe.2025.101234 PMC11849121 39995490

[18] Bossuyt PM, Irwig L, Craig J, Glasziou P. Comparative accuracy: assessing new tests against existing diagnostic pathways. BMJ. 2006;332(7549):1089–92. 10.1136/bmj.332.7549.1089 PMC1458557 16675820

[19] Whitehead AL, Julious SA, Cooper CL, Campbell MJ. Estimating the sample size for a pilot randomised trial to minimise the overall trial sample size for the external pilot and main trial for a continuous outcome variable. Stat Methods Med Res. 2016;25(3):1057–73. 10.1177/0962280215588241 PMC4876429 26092476

[20] Virk A, Strasburg AP, Kies KD, Donadio AD, Mandrekar J, Harmsen WS, et al. Rapid multiplex PCR panel for pneumonia in hospitalised patients with suspected pneumonia in the USA: a single-centre, open-label, pragmatic, randomised controlled trial. Lancet Microbe. 2024;5(12):100928. 10.1016/S2666-5247(24)00170-8 39426396

[21] Joung MK, Lee JA, Moon SY, Cheong HS, Joo EJ, Ha YE, et al. Impact of de-escalation therapy on clinical outcomes for intensive care unit-acquired pneumonia. Crit Care. 2011;15(2):R79. 10.1186/cc10072 PMC3219332 21366903

[22] Warreman EB, Lambregts MMC, Wouters RHP, Visser LG, Staats H, van Dijk E, et al. Determinants of in-hospital antibiotic prescription behaviour: a systematic review and formation of a comprehensive framework. Clin Microbiol Infect. 2019;25(5):538–45. 10.1016/j.cmi.2018.09.006 30267927

[23] Peri AM, Chatfield MD, Ling W, Furuya-Kanamori L, Harris PNA, Paterson DL. Rapid Diagnostic Tests and Antimicrobial Stewardship Programs for the Management of Bloodstream Infection: What Is Their Relative Contribution to Improving Clinical Outcomes? A Systematic Review and Network Meta-analysis. Clin Infect Dis. 2024;79(2):502–15. 10.1093/cid/ciae234 PMC11327801 38676943

[24] Enne VI, Stirling S, Barber JA, High J, Russell C, Brealey D, et al. INHALE WP3, a multicentre, open-label, pragmatic randomised controlled trial assessing the impact of rapid, ICU-based, syndromic PCR, versus standard-of-care on antibiotic stewardship and clinical outcomes in hospital-acquired and ventilator-associated pneumonia. Intensive Care Med. 2025;51(2):272–86. 10.1007/s00134-024-07772-2 PMC11903508 39961847

[25] Ling L, Lai CKC, Rhee C. Bacterial multiplex polymerase chain reaction tests for the diagnosis and management of pneumonia: ready for prime time? Thorax. 2025;80(11):862–72. 10.1136/thorax-2024-222297 40473414

[26] Di Pasquale MF, Sotgiu G, Gramegna A, Radovanovic D, Terraneo S, Reyes LF, et al. Prevalence and Etiology of Community-acquired Pneumonia in Immunocompromised Patients. Clin Infect Dis. 2019;68(9):1482–93. 10.1093/cid/ciy723 PMC6481991 31222287

[27] Contier J, Platon L, Benchabane N, Tchakerian S, Herman F, Mollevi C, et al. Diagnostic performance of Pneumonia multiplex PCR in critically ill immunocompromised patients. Crit Care. 2025;29(1):310. 10.1186/s13054-025-05528-y PMC12272964 40676679

