# Supplementary material for: Impact of rapid pathogen detection in ICU patients with suspected pneumonia on antimicrobial therapy: randomized, controlled open-label feasibility trial (IRISPAT-1)
Source: BMC Pulm Med. 2026 Aug 12;26:537. doi: 10.1186/s12890-026-04596-2 (PMC13746397; doi:10.1186/s12890-026-04596-2)
Supplement: Supplementary file 1 [file 12890_2026_4596_MOESM1_ESM.docx]

**Supplementary Methods**

**Antibiotic Treatment Algorithm**

**BioFire® FilmArray® Pneumonia Panel**

|  | **BioFire FilmArray Pneumonia Panel** |
| --- | --- |
| **Viruses** | Adenovirus  Coronavirus  Human metapneumovirus  Human rhinovirus/enterovirus  Influenza A virus  Influenza B virus  Parainfluenza virus  Respiratory syncytial virus |
| **Bacteria** | *Acinetobacter calcoaceticus-baumannii complex*  *Enterobacter cloacae complex*  *Escherichia coli*  *Haemophilus influenzae*  *Klebsiella aerogenes*  *Klebsiella oxytoca*  *Klebsiella pneumoniae group*  *Moraxella catarrhalis*  *Proteus spp.*  *Pseudomonas aeruginosa*  *Serratia marcescens*  *Staphylococcus aureus*  *Streptococcus agalactiae*  *Streptococcus pneumoniae*  *Streptococcus pyogenes*  *Chlamydia pneumoniae*  *Legionella pneumophila*  *Mycoplasma pneumoniae* |
| **Resistance Genes** | IMP  KPC  NDM  OXA-48-like  VIM  CTX-M  mecA/C and MREJ |

**BioFire® FilmArray® Pneumonia Panel Guided Antimicrobial Guidelines**

| **Pathogen** | **No Resistance Gene** | **CTXM** | **mecA/C-MREJ** | **KPC/VIM/IMP/NDM *** | **OXA-48 *** |
| --- | --- | --- | --- | --- | --- |
| *Acinetobacter calcoaceticus-baumannii* complex | Ampicillin/sulbactam or Cefoperazone/sulbactam | Meropenem |  | Ceftazidime/avibactam +/- Aztreonam or Cefiderocol |  |
| *Enterobacter cloacae* complex | Cefepime or Meropenem | Meropenem |  |  | Ceftazidime/avibactam or Cefiderocol |
| *Escherichia coli* | Ceftriaxone | Meropenem |  |  |  |
| *Haemophilus influenzae* | Amoxicillin/clavulanic acid or Ceftriaxone |  |  |  |  |
| *Klebsiella aerogenes* | Cefepime or Meropenem |  |  |  | Ceftazidime/avibactam or Cefiderocol |
| *Klebsiella oxytoca* | Ceftriaxone | Meropenem |  | Ceftazidime/avibactam +/- Aztreonam or Cefiderocol |  |
| *Klebsiella pneumoniae* group | Ceftriaxone | Meropenem |  |  |  |
| *Moraxella catarrhalis* | Ceftriaxone |  |  |  |  |
| *Proteus* spp. | Ceftriaxone | Meropenem |  | Ceftazidime/avibactam +/- Aztreonam or Cefiderocol | Ceftazidime/avibactam or Cefiderocol |
| *Pseudomonas aeruginosa* | Piperacillin/tazobactam or Ceftazidime | Meropenem |  |  |  |
| *Serratia marcescens* | Piperacillin/tazobactam or Ceftriaxone or Meropenem | Meropenem |  |  | Ceftazidime/avibactam or Cefiderocol |
| *Staphylococcus aureus* | Cloxacillin or Cefazolin |  | Vancomycin or Daptomycin or Linezolid or Ceftaroline |  |  |
| *Streptococcus agalactiae* | Ampicillin or Ceftriaxone |  |  |  |  |
| *Streptococcus pneumoniae* | Amoxicillin/clavulanic acid or Ceftriaxone |  |  |  |  |
| *Streptococcus pyogenes* | Penicillin or Ampicillin or Ceftriaxone |  |  |  |  |
| *Chlamydia pneumoniae* | Doxycycline or Azithromycin |  |  |  |  |
| *Legionella pneumophila* | Levofloxacin and Azithromycin |  |  |  |  |
| *Mycoplasma pneumoniae* | Doxycycline or Minocycline |  |  |  |  |

*Consultation with Microbiology/Infectious Diseases advised
