# Supplementary material for: Impact of rapid pathogen detection in ICU patients with suspected pneumonia on antimicrobial therapy: randomized, controlled open-label feasibility trial (IRISPAT-1)
Source: BMC Pulm Med. 2026 Aug 12;26:537. doi: 10.1186/s12890-026-04596-2 (PMC13746397; doi:10.1186/s12890-026-04596-2)
Supplement: Supplementary file 2 [file 12890_2026_4596_MOESM2_ESM.docx]

**Supplementary Table 1 Diagnosis, Causative Pathogen and Appropriate Antibiotics**

| **Study ID** | **Group** | **Diagnosis** | **Causative Respiratory Pathogen** | **Extra-pulmonary Infections** | **Empirical Antibiotics** | **Appropriate Antibiotics** |
| --- | --- | --- | --- | --- | --- | --- |
| **1** | Intervention | CAP | Influenza A |  | Piperacillin/tazobactam Minocycline | Stop antibiotics |
| **2** | Intervention | CAP | Influenza A |  | Ceftriaxone  Minocycline | Ceftriaxone |
| **3** | Control | CAP | *Streptococcus pneumoniae*, *Staphylococcus aureus*, Influenza A |  | Ceftriaxone  Minocycline  Vancomycin | Ceftriaxone plus clarithromycin |
| **4** | Control | CAP | Negative | *Clostridium difficile* colitis | Piperacillin/tazobactam Minocycline | Ceftriaxone plus vancomycin plus metronidazole |
| **5** | Intervention | Acute Pulmonary Edema | Negative | *Salmonella enterica* gastroenteritis | Ceftriaxone  Minocycline | Ceftriaxone |
| **6** | Control | Acute Pulmonary Edema | Negative |  | Piperacillin/tazobactam | Stop antibiotics |
| **7** | Intervention | CAP | *Staphylococcus aureus*, *Group B Streptococcus*, *Hemophilus influenzae*, *Moraxella spp* |  | Ceftriaxone  Minocycline | Ceftriaxone |
| **8** | Control | CAP | Human metapneumovirus |  | Ceftriaxone  Minocycline | Stop antibiotics |
| **9** | Intervention | VAP | *Klebsiella pneumoniae*, *Klebsiella aerogenes*, *Staphylococcus aureus* |  | Piperacillin/tazobactam | Cefepime or meropenem |
| **10** | Control | CAP | *Actinomyces spp* |  | Ceftriaxone  Minocycline | Penicillin |
| **11** | Control | VAP | Negative |  | Piperacillin/tazobactam | Piperacillin/tazobactam |
| **12** | Intervention | HAP | Negative |  | Meropenem | Piperacillin/tazobactam |
| **13** | Control | CAP | *Staphylococcus aureus*, *Hemophilus influenzae*, Coronavirus |  | Ceftraixone  Minocycline  Vancomycin | Ceftriaxone |
| **14** | Intervention | VAE | Negative |  | Piperacillin/tazobactam | Stop antibiotics |
| **15** | Intervention | VAP | *Staphylococcus aureus*, *Moraxella app*, *Hemophilus influenzae* |  | Piperacillin/tazobactam | Ceftriaxone |
| **16** | Control | CAP | *Acinetobacter baumannii* |  | Meropenem | Ampicillin/sulbactam |
| **17** | Control | CAP | *Hemophilus influenzae*, *Staphylococcus aureus*, *Klebsiella pneumoniae,* Human metapneumovirus |  | Ceftraixone  Minocycline | Ceftriaxone |
| **18** | Intervention | VAE | Negative |  | Piperacillin/tazobactam | Stop antibiotics |
| **19** | Control | CAP | *Moraxella spp*, *Proteus spp*, *Morganella morganii*, Respiratory Syncytial Virus |  | Piperacillin/tazobactam  Minocycline  Linezolid | Ceftriaxone or Cefepime |
| **20** | Intervention | Massive Hemoptysis from Lung Cancer | Negative |  | Ceftriaxone  Minocycline | Stop antibiotics |
| **21** | Intervention | CAP and Toxic Shock Syndrome | *Hemophilus influenzae*, *Klebsiella pneumoniae*, *Staphylococcus aureus*, Streptococcus pyogenes, Influenza A |  | Meropenem  Minocycline | Ceftriaxone plus linezolid/clindamycin |
| **22** | Control | HAP | Negative |  | Piperacillin/tazobactam | Piperacillin/tazobactam |
| **23** | Intervention | Massive Hemoptysis from Bronchiectasis | *Hemophilus influenzae* |  | Ceftriaxone  Minocycline | Stop antibiotics |
| **24** | Control | CAP and  Acute Pulmonary Edema | Negative |  | Piperacillin/tazobactam | Ceftriaxone |
| **25** | Control | CAP | *Legionella pneumophila*, *Staphylococcus aureus*, *Klebsiella aerogenes* |  | Ceftriaxone  Minocycline | Cefepime or meropenem plus levofloxacin or clarithromycin or minocycline |
| **26** | Control | HAP and  Acute Pulmonary Edema | Negative |  | Meropenem  Minocycline | Piperacillin/tazobactam |
| **27** | Intervention | VAP | *Staphylococcus aureus*, *Acinetobacter baumannii* |  | Piperacillin/tazobactam | Ampicillin/sulbactam |
| **28** | Intervention | CAP | SARS-CoV-2 |  | Piperacillin/tazobactam  Minocycline | Ceftriaxone |
| **29** | Intervention | VAP | *Serratia spp.* |  | Piperacillin/tazobactam | Ceftriaxone or cefepime or meropenem |
| **30** | Control | CAP | *Pseudomonas aeruginosa*, *Mycobacterium tuberculosis* |  | Piperacillin/tazobactam  Minocycline | Piperacillin/tazobactam plus anti-tuberculosis medications |
| **31** | Control | HAP | *Enterobacter cloacae*, *Human metapneumovirus* |  | Piperacillin/tazobactam  Minocycline | Cefepime or meropenem |
| **32** | Intervention | VAP | *Acinetobacter baumannii*, *Klebsiella pneumoniae*, *Staphylococcus aureus* |  | Meropenem | Ampicillin/sulbactam |
| **33** | Intervention | CAP | Negative |  | Meropenem  Minocycline | Ceftriaxone |
| **34** | Control | CAP | *Legionella pneumophila* |  | Piperacillin/tazobactam  Minocycline | Levofloxacin or clarithromycin or minocycline |
| **35** | Intervention | Asthma and  Upper Respiratory Tract Infection | Influenza A |  | Co-amoxiclav | Stop antibiotics |
| **36** | Control | CAP and  Acute Pulmonary Edema | *Staphylococcus aureus* |  | Ceftriaxone  Minocycline | Ceftriaxone |
| **37** | Control | VAP | *Staphylococcus aureus* |  | Cefoperazone/sulbactam | Ceftriaxone |
| **38** | Intervention | VAP | *Escherichia coli* |  | Meropenem | Ceftriaxone |
| **39** | Control | Burn Injury and Acute Pulmonary Edema | Negative |  | Piperacillin/tazobactam | Stop antibiotics |
| **40** | Intervention | CAP | *Hemophilus influenzae*, Rhinovirus |  | Ceftriaxone  Minocycline | Ceftriaxone |

Diagnosis ,causative pathogens (respiratory and extra-pulmonary) and appropriate antibiotics as determined by the Antibiotic Stewardship Review Panel. CAP, community-acquired pneumonia; HAP, healthcare-associated pneumonia; VAP, ventilator-associated pneumonia. Patients with only viral pathogens identified may still require antibiotics if there was concern of bacterial component to pneumonia.

**Supplementary Table 2 Diagnostic Performance**

|  | **Sensitivity** | **Specificity** | **Positive Predictive Value** | **Negative Predictive Value** | **AUC** |
| --- | --- | --- | --- | --- | --- |
| **mPCR and standard care** | 0.85 (0.75-1.00) | 0.91 (0.71-0.96) | 0.96 (0.88-1.00) | 0.73 (0.58-0.92) | 0.88 (0.78-0.99) |
| **Standard care alone** | 0.45 (0.27-0.65) | 0.92 (0.75-1.00) | 0.92 (0.78-1.00) | 0.44 (0.35-0.55) | 0.69 (0.56-0.82) |

Diagnostic performance of rapid mPCR in addition to standard care was assessed as an “add on test” and compared to standard care alone (18). The causative respiratory pathogen as determined by the antimicrobial stewardship review panel was used as the gold standard. Detection of colonizing pathogens were classified as false positive detection. mPCR, multiplex PCR. AUC, area under the receiver operating characteristic curve.
